# Characterization of Tumor Microenvironment and Prognosis of Regulatory T cells-Related Subtypes

**DOI:** 10.2174/0109298673375015250513094405

**Published:** 2025-07-10

**Authors:** Xinwei Li, Meiyun Nie, Keke Yang, Xiaodong Qi, Xiong Wan, Ling Yang

**Affiliations:** 1 Department of Geriatrics, Shanghai Fourth People's Hospital affiliated with Tongji University, Shanghai, China;; 2 Key Laboratory of Systems Health Science of Zhejiang Province, School of Life Science Hangzhou Institute for Advanced Study, University of Chinese Academy of Sciences, Hangzhou, China;; 3 Key Laboratory of Space Active Opto-Electronics Technology of the Chinese Academy of Sciences, Chinese Academy of Sciences, Shanghai Institute of Technical Physics, Shanghai, China

**Keywords:** Tumor microenvironment, immune infiltration, prognostic model, immunosuppressive cells, survival analysis, precision oncology

## Abstract

**Introduction:**

Regulatory T cells (Tregs) play an important role in the tumor microenvironment (TME). Currently, there have been no studies of Treg-related genes (TRGs) in lung adenocarcinoma (LUAD).

**Methods:**

We integrated the Cancer Genome Atlas (TCGA) dataset with the Gene Expression Omnibus (GEO) dataset and divided the TCGA-GEO dataset patient samples into different cohorts by unsupervised clustering analysis based on the expression of TRGs in LUAD. By analyzing the TME characteristics of different cohorts, we assessed immune cell infiltration and function. In addition, we constructed Cox risk proportional regression models based on TRGs to predict patient prognosis.

**Results:**

The results of unsupervised cluster analysis classified the TCGA-GEO dataset as “immune desert”, “immune evasion” and “immune inflammation”. Moreover, there was a significant survival differential among the three cohorts (*p*-value < 0.05). Based on the expression of 61 TRGs in LUAD, we screened TFRC, CTLA4, IL1R2, NPTN NPTN and METTL7A to construct a Cox risk proportional regression model to divide the TCGA-GEO dataset into a training cohort and a test cohort. Survival was significantly worse in the high-risk group than in the low-risk group in both the training and test cohorts (*p*-value < 0.05). Finally, the nomogram scoring system constructed by integrating the model risk scores with clinical parameters can well predict the 1, 3 and 5 year survival of patients.

**Conclusion:**

In conclusion, based on our analysis of the TRGs of LUAD patients, we can classify the patient TME into different immune statuses, which provides insights into adopting appropriate treatment regimens for different patients.

## 1 INTRODUCTION

Lung cancer is a leading cause of cancer-related mortality worldwide, posing a significant public health challenge. According to GLOBOCAN 2022, more than 2.2 million new cases of lung cancer are diagnosed annually, of which lung adenocarcinoma (LUAD) has become the most common pathological subtype [1]. Although the current advances in early screening methods, the development of new therapeutic agents and the clinical application of new treatments have improved the five-year survival rate of patients with lung cancer [2-5]. However, due to the highly aggressive and insidious nature of lung cancer, the 5-year survival rate for lung cancer patients is less than 20% and it kills approximately 1.76 million people worldwide each year [6-8]. Therefore, it is still necessary to find the appropriate treatment to improve the survival rate of patients.

Regulatory T cells (Tregs), discovered by Sakaguchi in 1995 [9], are a subpopulation of CD4^+^ T cells that have significant immunosuppressive effects and play an important role in maintaining the body’s immune homeostasis [10]. The physiological functions of Tregs include immune tolerance, contributing to the chronicity of the inflammatory response and immunosuppressive effects [11-14]. Studies have shown that immune cells can influence tumor progression by affecting the tumor microenvironment (TME) [12, 15, 16]. Tregs can inhibit the development and activation of effector cells that recognize their own tumor cells and play an important role in mediating the body’s immune tolerance to tumors [12, 17, 18]. In addition, the number of Tregs was negatively correlated with the prognosis of tumors [19]. Thus, Tregs play an important role in the regulation of tumor TME. Based on immune infiltration characteristics, the TME can be classified into three major phenotypes: Immune inflammation (“hot tumors”) - characterized by high immune cell infiltration and an active anti-tumor immune response. Immune evasion (“immune excluded”) - where immune cells are present but unable to penetrate the tumor core due to stromal barriers. Immune desert (“cold tumors”) - lacking effective immune infiltration and showing immune tolerance [20-22]. Understanding the functional role of Tregs and their associated genes (TRGs) in LUAD is crucial for optimizing immune-based therapeutic strategies [23]. However, no prior studies have comprehensively investigated the genomic landscape and prognostic significance of TRGs in LUAD.

In this study, we conducted a systematic investigation of TRGs in LUAD by integrating The Cancer Genome Atlas (TCGA) and Gene Expression Omnibus (GEO) datasets. Using unsupervised clustering analysis, we classified LUAD samples into distinct immune phenotypes based on TRG expression patterns. We further explored the TME composition, immune cell infiltration, and pathway enrichment within these subtypes. Additionally, we developed a TRG-based Cox regression model to predict 1-, 3-, and 5-year patient survival, aiding in personalized therapeutic decision-making. By elucidating the clinical significance and prognostic value of TRGs, our study provides novel insights into LUAD immune regulation and potential targets for immunotherapy.

## 2 MATERIALS AND METHODS

### 2.1 Acquisition and Analysis of Public Database Datasets

The LUAD dataset included in this study was obtained from the GEO (dataset: GSE68465; tumor = 442; https://www.ncbi.nlm.nih.gov/) and TCGA (tumor = 535; normal = 59; https://portal.gdc.cancer.gov/). In order to integrate the TCGA and GEO datasets, we use the “SVA” R language package to eliminate the batch effect between the TCGA and GEO datasets through the ComBat method. Table **1** presents the information obtained for the TCGA-GEO dataset with complete clinical follow-up data and survival time >30 days. The Treg-related genes (TRGs) set was obtained from the study of Yin He and Jie Yu [24, 25], and the 61 TRGs co-expressed in the TCGA and GEO datasets were obtained for this study (Supplementary Table **1**). Hallmark pathway gene set files obtained from the MSigDB database (http://www.gsea-msigdb.org/gsea/login.jsp). In addition, somatic mutations and copy number variants (CNVs) data in the TCGA dataset were obtained from the UCSC Xena database (https://xena.ucsc.edu/). The “maftools” R language package was used to visualize the mutation information of patients in the TCGA dataset, and the “RCircos” R language package was used to mark the location of 61 TRGs in human chromosomes. Analysis of 61 TRGs differentially expressed in LUAD and normal tissues was conducted by using the “limma” R language package.

### 2.2 Unsupervised Clustering Analysis

Based on the expression levels of 61 TRGs in the TCGA-GEO dataset, the patient samples were classified into different cohorts by the “ConsensusClusterPlus” R package. A consensus clustering algorithm was used to determine the number and stability of clusters [26]. The observations were repeatedly classified into a fixed number K (non-overlapping classes) by the K-Means algorithm. The K values were selected in accordance with the following characteristics: 1) Strong correlation among cohorts and weak correlation among different cohorts; 2) the cumulative distribution function curve at loci should increase gradually and smoothly; 3) the number of samples per cohort should not be too small.

### 2.3 Enrichment Analysis

Gene Set Variation Analysis (GSVA) [27] of Hallmark pathway gene sets was performed on the cohorts using the “GSVA” R package. The Estimation of Stromal and Immune Cells in Malignant Tumors using Expression Data (ESTIMATE) algorithm assesses the levels of stromal component, immune cell component and tumor purity in each sample using the “ESTIMATE” R package. Enrichment analysis of immune cells and functional gene sets in cohorts was conducted by using the single sample Gene Set Enrichment Analysis (ssGSEA) method.

### 2.4 Single-cell RNA Sequencing Data Acquisition and Processing

The single-cell RNA sequencing (scRNA-seq) data for lung cancer patients undergoing neoadjuvant immunotherapy combined with chemotherapy were obtained from the GEO database (GSE207422) [28]. Based on pathological response, patients were divided into two groups: major pathological response (MPR) group (residual viable tumor cells ≤10%) and non-major pathological response (NMPR) group (residual viable tumor cells >10%). Sample02, Sample07, Sample09, and Sample15 belonged to the NMPR group, while Sample03, Sample06, Sample11, and Sample14 belonged to the MPR group.

To integrate and correct batch effects, we used the “Harmony” R package. Further preprocessing, including quality control, normalization, visualization, dimensionality reduction, and clustering, was performed using the “Seurat” R package. The quality control criteria were as follows: 1) Total gene expression per cell ≤ 1,000; 2) Number of detected genes per cell ≤ 500; 3) Mitochondrial gene expression > 20%; 4) The FindNeighbors function (reduction = “harmony”, dims = 1:15) was applied to compute the neighborhood graph and assess cell similarity. The optimal clustering resolution was set to 0.5 based on clustering analysis.

### 2.5 Cell Subtype Annotation

Cell subpopulation annotation began by identifying marker genes for each cluster using the FindAllMarkers function. Next, clusters were automatically annotated using the “SingleR” R package, with the HumanPrimaryCellAtlasData database as a reference. Finally, the annotation was refined by incorporating previously published marker genes for cell subpopulations.

### 2.6 Construction of a Cox Risk Regression Model

Since the TCGA dataset has more complete clinicopathological parameters, we selected the TCGA dataset samples to construct the Cox risk regression model. The prognosis-related TRGs were first screened by univariate Cox analysis of 61 TRGs, and then the prognosis-related TRGs were screened for variables by the least absolute shrinkage and selection operator regression (LASSO) algorithm. Finally, the screened variables were subjected to stepwise multivariate Cox risk proportional regression analysis to construct a TRGs-related Cox risk model. The formula for constructing the Cox risk model is as follows:


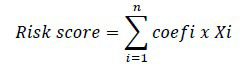


where “coefi” and “Xi” represent the coefficient and expression level of each TRGs, respectively. Based on the median score of the Cox risk model, the patient sample was divided into high- and low-risk groups and the “survival” R package was used to compare the survival differences between the high- and low-risk groups.

### 2.7 Assessment of Clinical Effectiveness of the Risk Score

Independent prognostic analysis was performed by integrating risk score with clinicopathological parameters using univariate and multivariate Cox analysis methods in LUAD. Then, the “rms” R package was used to integrate risk score with clinicopathological parameters to construct the nomogram survival prediction system [29] to predict 1-, 3- and 5-year survival rates of patients. In addition, calibration curves of the nomogram survival prediction system were plotted by using the “regplot” R package.

### 2.8 Statistical Analysis

The statistical analysis and visualization of data in this study were done by using R software (version 4.1.1). For quantitative data, the student’s t-test was used to evaluate the statistical significance of normally distributed variables, while the Wilcoxon rank-sum test was used to examine non-normally distributed variables. The log-rank test was used to compare data between the two groups, while the Kruskal-Wallis test was used to compare data between more than two groups.

## 3 RESULTS

### 3.1 Landscape Demonstration of TRGs Mutation in LUAD

**Fig. (1):**
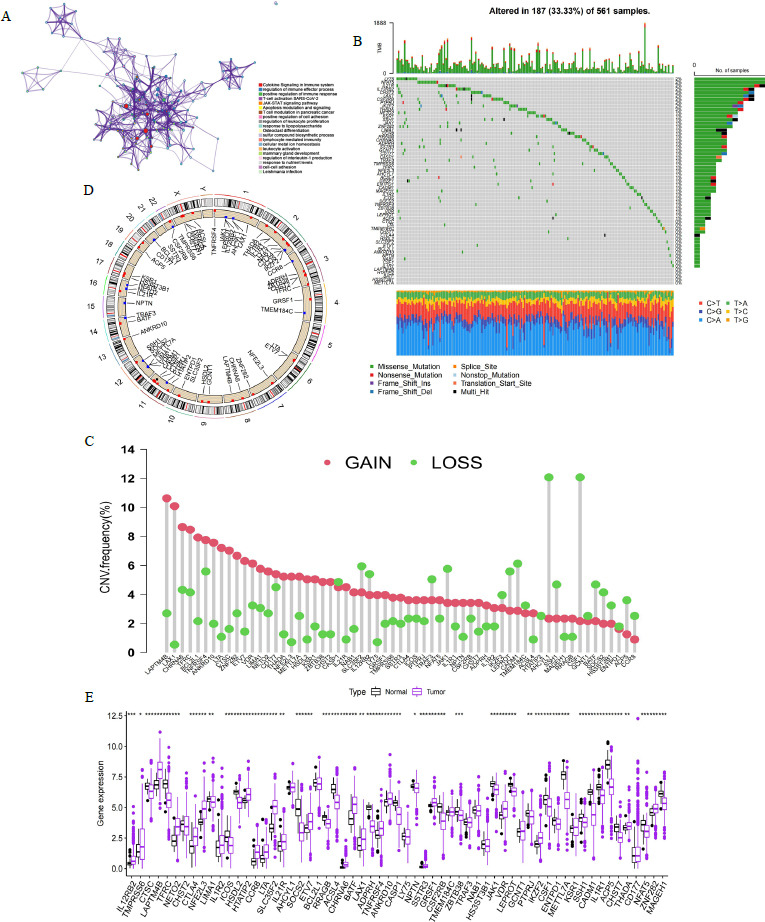
Mutation analysis of 61 TRGs in LUAD. (**A**) Functional enrichment analysis of 61 TRGs in the Metascape website. (**B**) Somatic mutations and (**C**) CNVs analysis of 61 TRGs in LUAD in TCGA dataset. (**D**) Location of 61 TRGs on human chromosomes. (**E**) Differential analysis of 61 TRGs in LUAD and normal tissues. *: *p*-value < 0.05; **: *p*-value < 0.01; ***: *p*-value < 0.001.

Functional enrichment analysis of 61 TRGs through the Metascape website (http://metascape.org/gp/index) revealed that this gene set is mainly involved in immune regulation, such as cytokine signaling in the immune system, regulation of immune effector processes and positive regulation of immune response (Fig. **1A**). Somatic mutation analysis based on the TCGA dataset indicated that 61 TRGs were mutated in 187 of the 561 LUAD (33.33%) samples, with missense mutations predominating and the highest proportion of mutations in LY75 (2%), NFAT5 (2%), IL21R (2%), IL12RB2 (2%), CHST2 (2%), LAX1 (2%), CSF2RB (2%), PTPRJ (2%), IKZF2 (2%) and THADA (2%) (Fig. **1B**). Further analysis revealed that CNVs were commonly present in these 61 TRGs and were predominantly copy number amplified, with LAPTM4B being the most frequently amplified and AHCYL1 and CSF1 being the most frequently deleted (Fig. **1C**). In addition, Fig. (**1D**) demonstrates the location of the CNVs of the 61 TRGs on the human chromosome. Differential analysis of 61 TRGs in LUAD revealed that 24 TRGs were significantly upregulated and 25 TRGs were significantly downregulated in tumor tissues compared to normal tissues. Altogether, 61 TRGs have significantly different mutation and expression levels in LUAD, which may have a potential impact on LUAD development (Fig. **1E**).

### 3.2 Classification of LUAD Patient Samples Based on TRGs

**Fig. (2):**
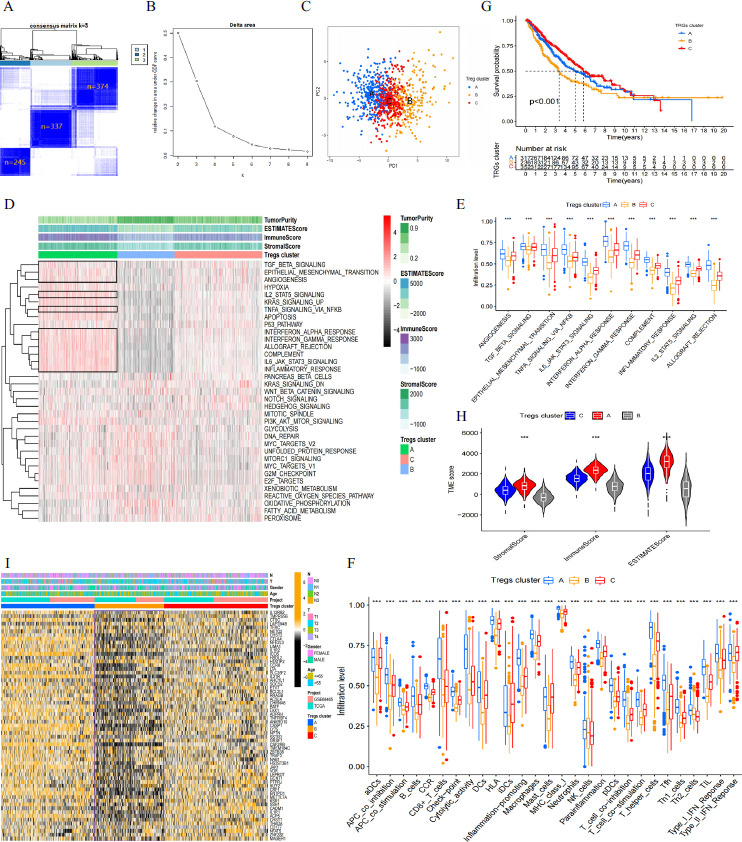
Unsupervised clustering analysis and cohort characterization of TCGA-GEO dataset in LUAD. (**A**, **B**) Results of unsupervised clustering analysis of TCGA-GEO dataset (K = 3). (**C**) PCA analysis of TCGA-GEO dataset based on three cohorts. (**D**) Heatmap of enrichment analysis of Hallmark pathway gene sets and TME components in three cohorts. (**E**) Differential enrichment analysis of immune and stroma-related signaling pathways in three cohorts (*p*-value < 0.05). (**F**) Differential enrichment analysis of immune cells and functional gene sets in three cohorts (*p*-value < 0.05). (**G**) Analysis of survival differences among three cohorts in LUAD (*p*-value < 0.05). (**H**) Differential analysis of immune and stromal components in TME in three cohorts. (**I**) Heatmap of TRGs expression in LUAD enrichment analysis in three cohorts (*p*-value < 0.05). ***: *p*-value < 0.001.

To explore the impact of TRGs on LUAD progression, we performed typing on the TCGA-GEO dataset by unsupervised clustering methods. Based on the results of the analysis, we divided the patient sample into three cohorts (K = 3; Cohort A = 337; cohort B = 245; cohort C = 374; Figs. **2A** and **B**). Principal component analysis (PCA) showed that the three cohorts divided according to unsupervised clustering could distinguish the TCGA-GEO dataset samples well (Fig. **2C**). Further we explored the TME characteristics of the three cohorts in LUAD by multidimensional enrichment analysis. The enrichment heatmap illustrated that cohort B is downregulated in immune-regulated signaling pathways (IL2 STAT5 signaling, IL6 JAK STAT3 signaling, inflammatory response signaling, interferon α/γ response signaling, complement signaling, allograft rejection and TNF alpha *via* NF-kB signaling) and stromal signaling pathways (TGF β signaling, epithelial-mesenchymal transition signaling and angiogenesis signaling) (Fig. **2D**). However, cohorts A and C are upregulated in these signals and Cohort A is upregulated to a greater extent than cohort C (Fig. **2D**). In addition, the three cohorts were significantly differentially enriched in these signaling pathways (*p*-value < 0.05; Fig. **2E**). Consistently, immune cell and functional enrichment analysis indicated that Cohort A was the most enriched and cohort B the least enriched, and there was significant differentiation among the three cohorts (*p*-value < 0.05; Fig. **2F**).

Survival analysis indicated significant differences among the three cohorts (*p*-value < 0.05; Fig. **2G**). Cohort B has the worst prognosis. Interestingly, the enrichment of immune signals, immune cells and immune functions was higher in Cohort A than in Cohort C, while survival was better in Cohort C than in Cohort A (Fig. **2G**). We speculate that immune evasion is present in the TME of Cohort A and that the tumor stromal component prevents immune cells from infiltrating the tumor parenchyma to function (Fig. **2D**-**H**). In addition, CD8^+^ T cells and immune checkpoints were expressed at higher levels in Cohort A compared to Cohort C, and these genes were expressed at the lowest levels in Cohort B (Fig. **2F**). According to Hegde’s classification of TME, we concluded that TME in Cohort B was consistent with “immune desert”, Cohort A with “immune evasion”, and Cohort C with “immune inflammation” [20]. Finally, the TRGs expression enrichment heatmap results were consistent with the immune signaling pathway, immune cell infiltration and immune cell function enrichment results, with Cohort A gene expression being the highest while Cohort B expression was the lowest (Fig. **2I**). This implies that TRGs can modulate the TME status of patients with LUAD to influence the prognosis of patients.

### 3.3 ScRNA-seq Reveals Different Functional States of Tregs

**Fig. (3):**
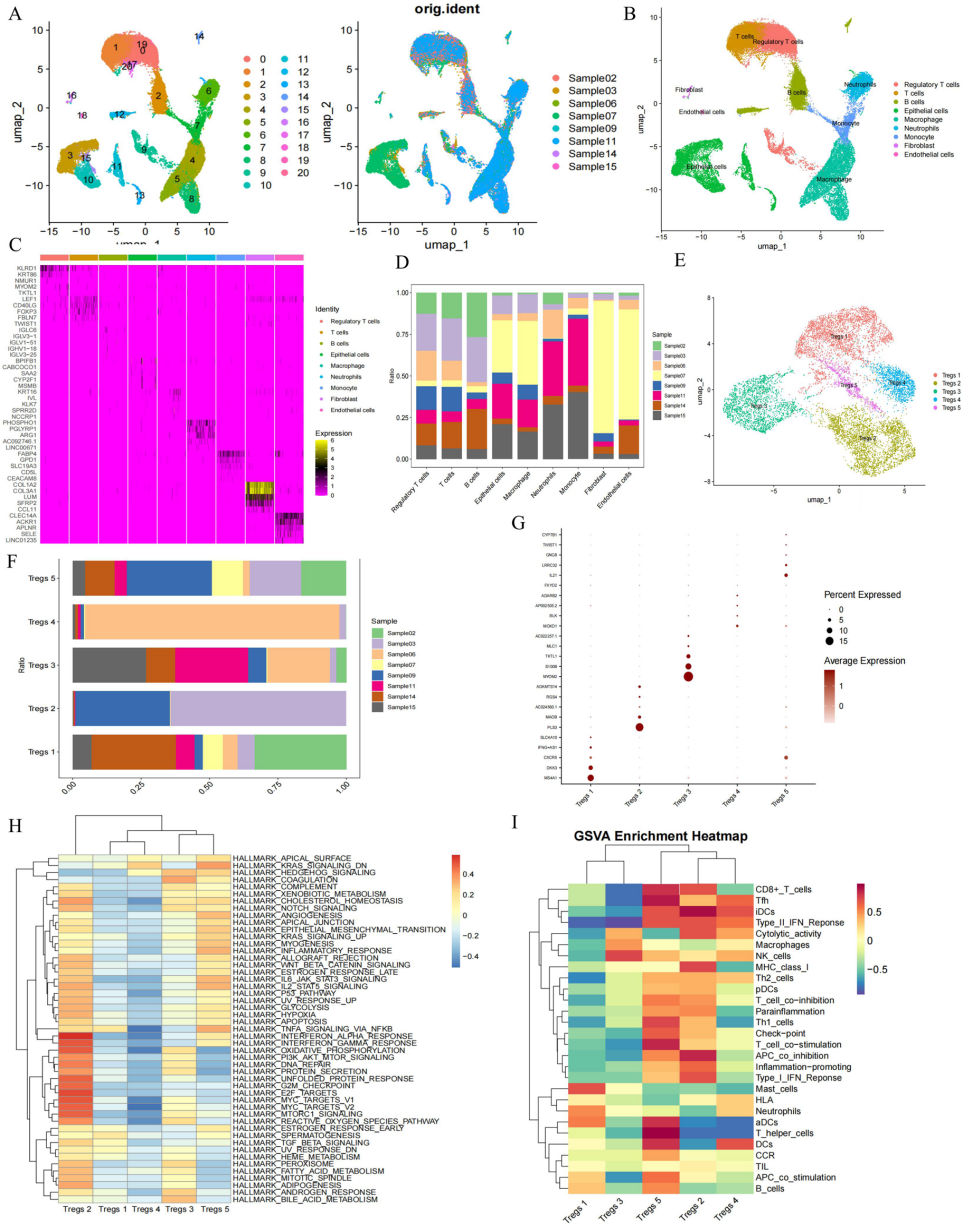
ScRNA-seq analysis of Tregs in LUAD. (**A**) UMAP visualization of scRNA-seq clustering. (**B**) Cell-type annotation. Different cell populations were identified. (**C**) Heatmap of marker gene expression across different cell clusters. (**D**) Proportional distribution of cell types across different patient samples. (**E**) UMAP visualization of Tregs subpopulations.(**F**) Proportional distribution of Tregs subpopulations across different samples. (**G**) Dot plot showing marker gene expression in each Tregs subpopulation. (**H**) GSVA enrichment analysis of hallmark pathways in Tregs subpopulations. (**I**) GSVA enrichment heatmap of immune cell signatures and immune functions.

After quality control and data filtering, transcriptomics data from 52422 cells were included in this study. Dimensionality reduction and clustering of these cells resulted in 20 clusters (Fig. **3A**), which were annotated based on the expression of typical marker genes, identifying them as T cells, Tregs, macrophages, epithelial cells, monocytes, neutrophils, B cells, fibroblasts, mast cells, and endothelial cells (Fig. **3B**). The heatmap presents the top 5 marker genes for each cell subpopulation (Fig. **3C**). Fig. (**3D**) shows the distribution of cell subpopulations across samples, reflecting TME heterogeneity. Similarly, there is significant heterogeneity in the proportions of cell subpopulations between the MPR and NMPR groups.

To further explore the heterogeneity of Treg functional states, we selected Treg subpopulations for further analysis. After dimensionality reduction and clustering, we identified five Treg subpopulations (Fig. **3E**-**G**). GSVA enrichment analysis showed that Tregs 1, Tregs 3, and Tregs 4 were downregulated in immune activation signaling pathways, such as interferon and interleukin signaling, consistent with the “immune desert” microenvironment. Tregs 1 and Tregs 5 exhibited upregulation in immune activation pathways; however, Tregs 1 showed significant upregulation in immune evasion signals like TGF-β, aligning with an “immune evasion” microenvironment, whereas Tregs 5 was classified as an “immune inflammatory” phenotype (Fig. **3H**). Additionally, enrichment analysis of immune cells and functional signals further supported these findings (Fig. **3I**). The scRNA-seq results were consistent with bulk RNA analysis, confirming that Tregs shape distinct immune states within the TME.

### 3.4 Prognostic Analysis of TRGs in LUAD

**Fig. (4):**
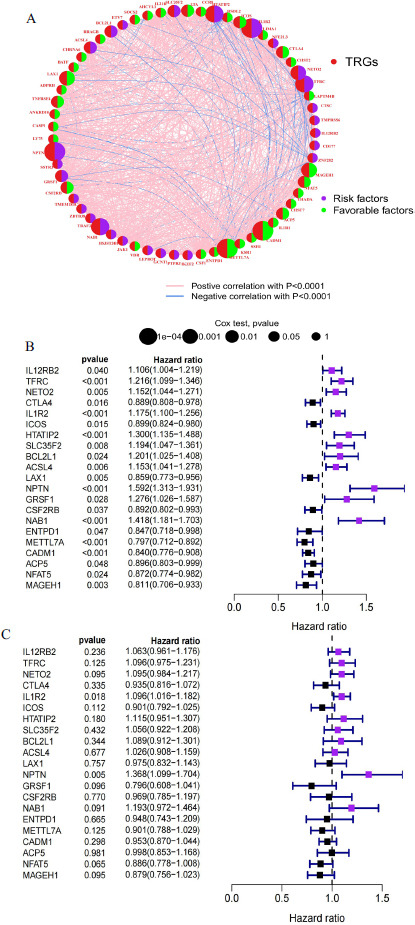
Prognostic analysis of TRGs in LUAD. (**A**) Interaction network of the 61 TRGs in LUAD. Circles in red represent TRGs, purple represents risk factors, green represents favorable factors, pink lines represent significant positive correlations, and blue lines represent significant negative correlations. (**B**) Univariate and (**C**) Multivariate Cox prognostic analysis of TRGs in LUAD (*p*-value < 0.05).

Further, we explored the prognostic significance of 61 TRGs in LUAD. In the TRGs regulatory network, the interactions among the 66 TRGs, their interconnections and their importance for the prognosis of patients with LUAD were comprehensively described (Fig. **4A**). Univariate Cox analysis demonstrated that 21 TRGs were prognostically significant in LUAD, with 11 genes associated with poor prognosis and 10 genes associated with good prognosis (Fig. **4B**). Multivariate Cox analysis revealed that IL1R2 and NPTN among these genes can be used as independent prognostic risk factors for LUAD (Fig. **4C**). Altogether, this suggested that TRGs are involved in regulating LUAD progression.

### 3.5 Construction of a TRGs-related Cox Risk Regression Model in LUAD

**Fig. (5):**
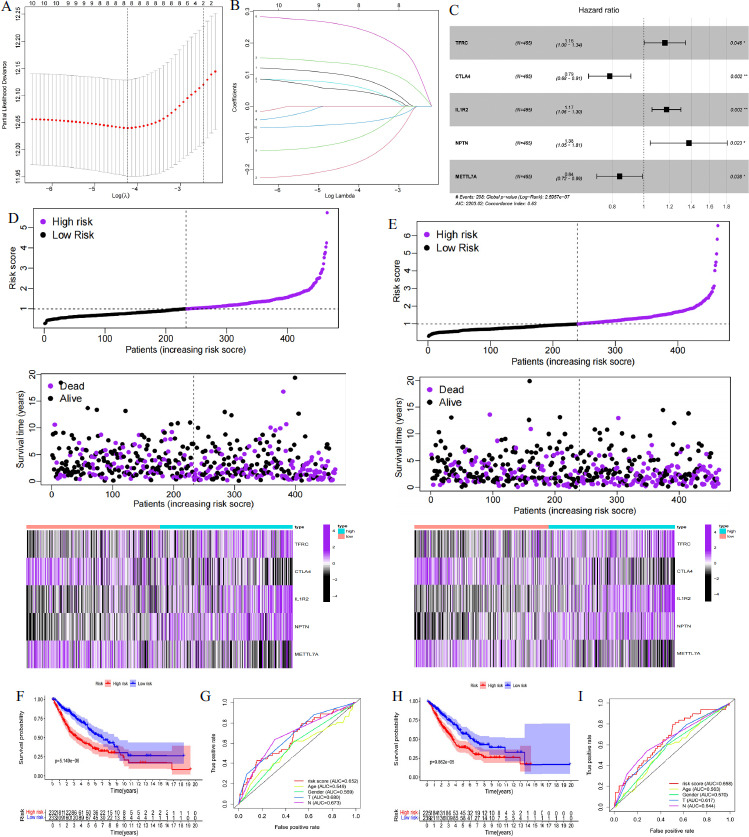
Construction of a TRGs-related Cox risk proportional regression model. (**A**, **B**) Screening of candidate genes for constructing Cox risk proportional regression model by LASSO method. (**C**) TFRC, CTLA4, IL1R2, NPTN NPTN and METTL7A were screened for the construction of the Cox risk proportional regression model. The training (**D**) and test (**E**) cohorts were divided into high- and low-risk groups based on the median values of the model risk scores. Analysis of survival differences between the high-risk and low-risk groups in the training cohort (**F**) and the ROC curves evaluate the accuracy of the predictions of the training cohort (**G**). Analysis of survival differences between the high-risk and low-risk groups in the test cohort (**H**) and ROC curves evaluate the accuracy of the predictions of the test cohorts (**I**) Divided by the model.

To better predict the prognosis of patients with LUAD, we constructed a Cox risk proportional regression model based on the expression levels of 61 TRGs in LUAD. According to the univariate Cox analysis of 61 TRGs, we screened five candidate target genes (TFRC, CTLA4, IL1R2, NPTN and METTL7A) for model construction by the LASSO method and divided the TCGA-GEO dataset into a training (n = 465) cohort and a test cohort (n = 464) (Fig. **5A**-**C**). Risk score = (coefficient TFRC × expression TFRC) + (coefficient CTLA4× expression CTLA4) + (coefficient IL1R2× expression IL1R2) + (coefficient NPTN × expression NPTN) + (coefficient METTL7A × expression METTL7A). Using the LUAD dataset from the starBase database (https://rnasysu.com/encori/index.php) [30], we analyzed the expression levels of key model genes in tumor and normal tissues. CTLA4 and IL1R2 were expressed at higher levels in tumor tissues compared to normal tissues, while NTPN, TFRC, and METTL7A showed lower expression in tumor tissues than in normal tissues. This indicates expression heterogeneity of the model genes between tumor and normal tissues (Supplementary Fig. **1A**). Furthermore, scRNA-seq analysis revealed differential expression of the model genes across different functional Treg subpopulations, suggesting their crucial role in regulating the immune state of Tregs (Supplementary Fig. **1B**).

The training cohort was divided into high- and low-risk groups based on the median risk score of the model, and survival analysis showed that the high-risk group survived significantly worse than the low-risk group (*p*-value < 0.05; Fig. **5D** - **F**). The ROC curve assessment showed a high accuracy (AUC = 0.652) of the training cohort survival assessment (Fig. **5G**). In addition, the high-risk group survived significantly worse than the low-risk group in the test cohort and also had higher accuracy (*p*-value < 0.05; AUC = 0.658; Figs. **5E**, **H** and **I**).

### 3.6 Assessment of Cox risk Regression Model Score Effectiveness

**Fig. (6):**
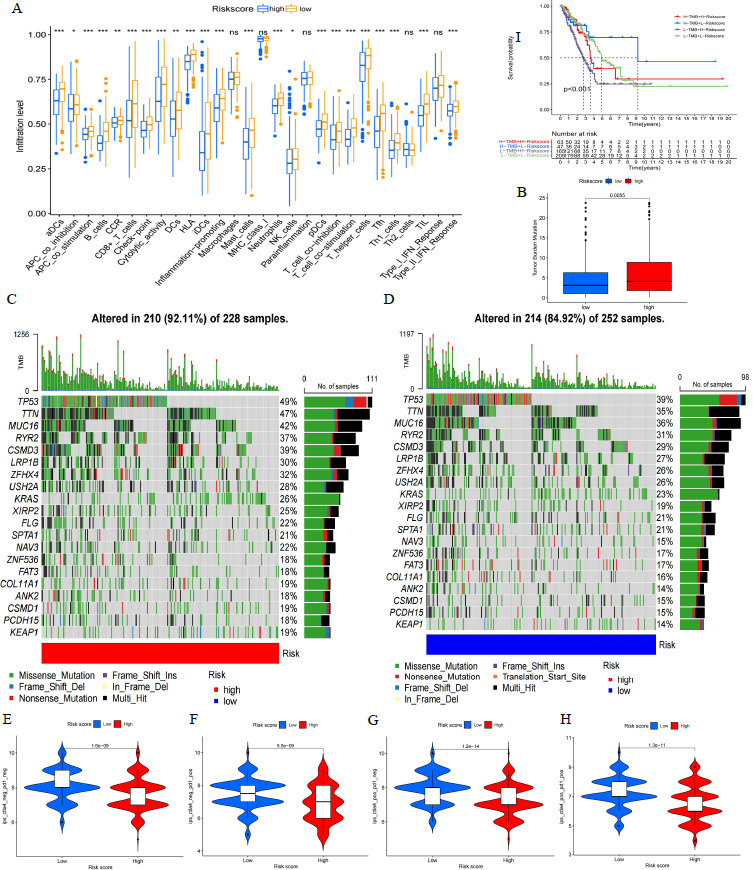
Analysis of the role of model score of risk in TME in patients with LUAD. (**A**) Immune cell and function enrichment analysis in the high- and low-risk groups of the model score (*p*-value < 0.05). (**B**) Analysis of TMB differences in high- and low-risk groups in the model score (*p*-value < 0.05). Statistics of somatic mutations in high- (**C**) and low-risk score groups (**D**) of the model score. (**E-H**) Evaluation of immunotherapy efficacy in PD-1 and CTLA4-positive or -negative patients (*p*-value < 0.05). (**I**) Survival analysis of somatic mutations combined with model risk score (*p*-value < 0.05).*: *p*-value < 0.05 ; **: *p*-value < 0.01; ***: *p*-value < 0.001.

Enrichment analysis of immune cells and function in the high- and low-scoring groups of the Cox risk proportional regression model revealed that immune cells and function were significantly worse in the high-risk group than in the low-risk group (*p*-value < 0.05; Fig. **6A**). Among them, CD8^+^ T cells and immune checkpoints were significantly higher in the low-risk scoring group than in the high-risk scoring group. Immunotherapy was significantly more effective in the low-risk score group than in the high-risk score group for patients with LUAD, regardless of whether they were positive or negative for PD-1 and CTLA4. Somatic mutation analysis revealed significantly higher TMB in the high-risk group than in the low-risk group (*p*-value < 0.05; Fig. **6B**). The somatic mutation statistics in the high- (92.11%) and low-risk groups (84.92%) are demonstrated in Figs. (**6C** and **D**). The highest proportion of TP53 mutations was found in both high- (49%) and low-risk groups (39%) (Figs. **6E**-**H**). Survival analysis revealed that significant differences were found among cohorts of somatic mutation integration risk scores and that patients with low-risk scores with high-mutation had the best survival and those with high-risk scores with low-mutation had the worst survival (*p*-value < 0.05; Fig. **6I**). Based on the above analysis, it was shown that high- and low-risk scores classified according to the model could well predict the prognosis of patients with LUAD.

**Fig. (7):**
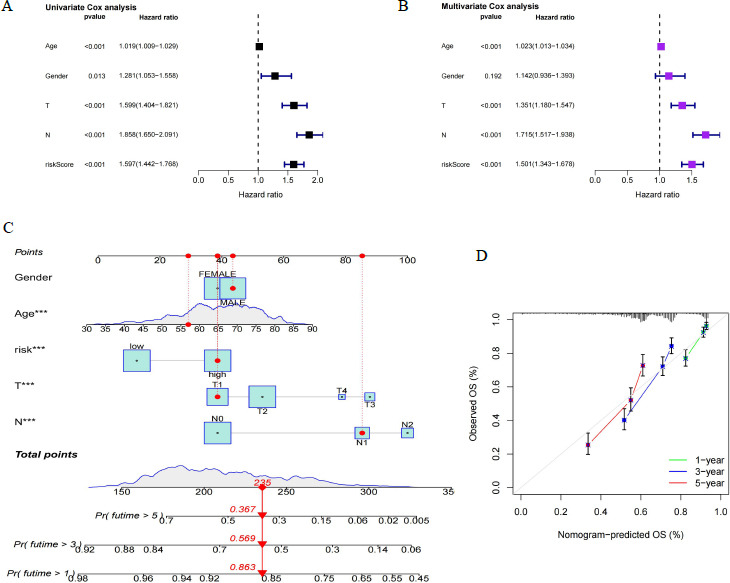
Prognostic prediction by Cox risk model risk score combined with clinical parameters. (**A**, **B**) Integration of model risk score with clinical parameters for univariate and multivariate Cox analysis. (**C**) Construction of nomogram survival prediction scoring system by integrating model risk score with clinical parameters. (**D**) Calibration curves to assess nomogram survival prediction. ***: *p*-value < 0.001.

Univariate Cox analysis revealed that age, sex, T, N, and risk score factors were associated with poor prognosis in LUAD (Fig. **7A**). Multivariate Cox analysis showed that age, T, N and risk score could be used as risk factors for independent prognosis of LUAD (Fig. **7B**). Therefore, the model risk assessment has good predictive power for patients with LUAD. Finally, we constructed a nomogram survival prediction scoring system incorporating model risk scores to predict 1-, 3-, and 5-year survival of patients (Fig. **7C**). The calibration curves showed a high accuracy of the model's predictive capability (Fig. **7D**).

## 4 DISCUSSION

Lung cancer has the highest mortality rate of all cancers and contributes to the global healthcare burden [6, 8, 31, 32]. The clinical application of immunotherapy has greatly improved the prognosis of patients with lung cancer and brought new hope to patients with lung cancer [5, 33-35]. However, not all patients can benefit from immunotherapy [34, 36]. There are many aspects of current research for patients with poor immune response, and Treg is one of the important factors in maintaining the body’s immune tolerance and plays an important role in tumor progression. In mouse melanoma and breast cancer, cancer cells produced by Treg expressing αvβ8 integrin β8 chains activate TGF-β signaling in tumors, thereby shaping the immunosuppressive state in TME [37]. Cancer-associated fibroblasts directly ligate and induce naive CD4^+^ T cells to become Treg in an antigen-specific manner to participate in pancreatic cancer TME immune evasion [38]. The density of PD-L1 + Tregs in lung cancer correlates with CD8^+^ T cell exhaustion in TME and affects TME immune status [39]. Therefore, research on Treg cells can help to change the TME status to enhance the effect of immunotherapy [40, 41].

In our present study, we divided the 61 TRGs in LUAD into three cohorts by performing unsupervised cluster analysis. By analyzing the TME of the three cohorts, we found that Cohort A was consistent with “immune evasion”, cohort B with “immune desert”, and cohort C with “immune inflammation”. The “immune desert” phenotype is characterized by a lack of immune cell infiltration and low expression of immune-related pathways (*e.g.*, MHC I, antigen presentation pathways) [20, 42, 43]. Tumors with this phenotype tend to exhibit high proliferation rates and genomic stability, which leads to a poor response to immune checkpoint inhibitors, such as anti-PD-1 or anti-CTLA4 [20, 42, 44]. These tumors may also have a low mutational burden, which contributes to a weak immune response [20]. For tumors with these phenotypes, combination therapies could be particularly effective. Targeting Tregs by using anti-CD25 antibodies or anti-CTLA4 monoclonal antibodies can help reduce Tregs-mediated immune suppression and improve immune cell infiltration [18, 45, 46]. The “immune evasion” phenotype is characterized by the presence of immune cells in the TME that are unable to exert effective anti-tumor responses due to barriers within the tumor stroma (*e.g.*, tumor-associated fibroblasts, extracellular matrix) [47, 48]. High levels of TGF-β signaling and the presence of myeloid-derived suppressor cells (MDSC) contribute to immune tolerance and dysfunction [49-51]. For tumors with these phenotypes, therapies targeting Tregs are crucial. The use of anti-CD25 antibodies or anti-CTLA4 monoclonal antibodies can help reduce Tregs and facilitate immune cell infiltration into the tumor, improving TME conditions [18, 45, 46]. Additionally, combining Treg-targeting therapies with agents that disrupt stromal barriers (such as TGF-β inhibitors) may further enhance the efficacy of immune checkpoint blockade and improve immune responses [52-54]. The “immune inflammation” phenotype is characterized by a high density of infiltrating immune cells, including TILs and effector T cells, indicating an active immune response in the TME [55]. However, even in this phenotype, Tregs and immune checkpoint molecules can inhibit effector T cell responses, leading to immune exhaustion [56]. In this phenotype, targeting Tregs to reduce their suppressive activity is critical [57]. Treatment strategies using anti-CCR4 antibodies can effectively deplete or inhibit Tregs, reducing their impact on effector T cells and restoring anti-tumor immunity [58, 59]. These therapies, when combined with immune checkpoint blockade, can help rejuvenate the immune response and enhance the effectiveness of immunotherapy.

Emerging research suggests that immune cell infiltration and spatial organization within the TME may be influenced not only by molecular and genetic mechanisms but also by biophysical and thermodynamic interactions [60, 61]. The Flory-Huggins interaction parameter, traditionally used in polymer physics to describe phase separation, has been proposed to explain Tregs-mediated immune suppression in tumors [62-64]. In immune desert phenotypes, high interfacial energy between Tregs and tumor cells could drive immune exclusion, akin to phase separation [65, 66]. In immune evasion phenotypes, intermediate Flory-Huggins parameters may favor Treg interactions with stromal components, forming immune-exclusion barriers [66, 67]. Conversely, immune inflammation phenotypes may reflect a well-mixed phase of Tregs and tumor cells, promoting immune equilibrium but suppressing effector T cell activation [67]. Applying thermodynamic models to Treg dynamics in LUAD TME offers a novel framework for understanding immune cell clustering, spatial heterogeneity, and therapy resistance, potentially optimizing immunotherapy strategies. These insights align with findings that immune checkpoint blockade efficacy may depend on biophysical constraints governing immune cell movement. Research has demonstrated that oncogenes and aberrant pathway signaling are central to the establishment of different states of TME in tumor patients [68]. The heatmap of 61 TRG expression in LUAD revealed that TRG expression was highest in Cohort A and lowest in Cohort B, indicating that TRGs can regulate TME immune status in LUAD. Therefore, we screened TFRC, CTLA4, IL1R2, NPTN and METTL7A TRGs based on 61 TRGs expressed in LUAD to construct a Cox risk proportional regression model. TFRC plays a critical role in mediating iron uptake, which is vital for tumor cell growth and immune modulation. Overexpression of TFRC in tumors has been linked to immune evasion by promoting tumor cell proliferation while inhibiting the infiltration and activation of immune cells within the TME [69]. CTLA4 is a well-known immune checkpoint receptor that inhibits T-cell activation, promoting immune tolerance and immune evasion within the TME [70, 71]. In LUAD, CTLA4 expression on Tregs contributes to a suppressive immune environment, preventing effective anti-tumor immune responses [72]. Targeting CTLA4 is a promising immunotherapeutic approach to enhance immune surveillance and tumor rejection [73]. IL1R2 acts as a decoy receptor for IL-1, modulating inflammatory responses by reducing IL-1 signaling. Upregulation of IL1R2 in LUAD can promote immune evasion by dampening the anti-tumor immune response, particularly in TME states characterized by immune suppression and inflammation [74]. NPTN is involved in nucleotide exchange and energy regulation, contributing to metabolic reprogramming in tumors [75]. Downregulation of NPTN in cancer may impair immune cell infiltration and T cell function, fostering an immune-suppressive environment and hindering anti-tumor immunity within the TME [76]. METTL7A regulates gene expression through methylation, influencing immune responses within the TME [77]. Altered METTL7A expression in LUAD has been associated with modulation of immune cell function by affecting the methylation of immune-related genes, thus playing a role in immune evasion and immune suppression within the tumor [78]. Based on the model risk score, we can assess the immune cells and function, TMB and immunotherapy effects in patients with LUAD. Therefore, studies targeting these five genes are important for improving the prognosis of patients with LUAD.

While our study provides valuable insights into the role of TRGs in LUAD and their potential for predicting patient survival, there are several limitations that need to be considered. First, our analysis is based on publicly available datasets (TCGA and GEO), which may have inherent biases and may not fully represent the diversity of LUAD patients in the broader population. Additionally, the lack of functional validation for the identified TRGs means that their exact mechanisms of action within the TME remain speculative and require further experimental studies. Furthermore, our study is retrospective in nature, which limits its ability to establish causality and the ability to generalize the findings to clinical practice. Although we performed robust bioinformatic analyses, future prospective studies incorporating patient-specific data and experimental models are needed to validate these findings. Finally, the model we developed for predicting patient prognosis based on TRGs is primarily dependent on gene expression data and does not incorporate other critical factors, such as genetic mutations or clinical variables that may further enhance its predictive accuracy.

## CONCLUSION

In summary, we divided patients with LUAD into distinct cohorts based on the expression of 61 TRGs and constructed prognostic models to assess individual patient survival. By evaluating the TME characteristics of these cohorts, we identified key molecular pathways associated with tumor progression and immune modulation. This classification approach enables the development of tailored therapeutic strategies, aligning with the principles of precision medicine. The study emphasizes the importance of incorporating immune phenotyping into clinical practice to refine treatment regimens for LUAD patients. Our findings highlight the potential of TRGs as biomarkers for prognosis and therapeutic targets, suggesting that strategies targeting immune regulation could significantly improve patient outcomes. However, further research, including functional validation and clinical trials, is needed to validate these findings and explore their potential in clinical settings. Overall, this study contributes to advancing personalized therapy for LUAD and enhancing our understanding of the role of immune cells in the tumor microenvironment.

## Figures and Tables

**Table 1:** Clinical information on LUAD patients in the TCGA and GEO datasets.

**Clinical Information**	**TCGA Dataset**	**GSE68465**
Number of patients	508	439
Platform	Illumina RNAseq	Affymetrix Human Genome U133A Array
Gender	-	-
Female	272	218
Male	236	221
Age (years)	-	-
Range	33-88	33-87
unknown	19	0
Stage	-	-
I	272	130
II	120	34
III	83	26
IV	25	4
unknown	8	0
T	-	-
T1	167	150
T2	275	248
T3	45	28
T4	18	11
unknown	3	2
N	-	-
N0	327	297
N1	96	87
N2	72	52
N3	2	0
unknown	11	3
M	-	-
M0	342	——
M1	24	——
unknown	142	——
Follow-up time (days)	-	-
Range	33-7248	50-5712
Survival	-	-
Death	183	206
Alive	325	233

## Data Availability

The raw data used in this study were downloaded from GEO: https://www.ncbi.nlm.nih.gov/; TCGA: https://portal.gdc.cancer.gov/; MSigDB: http://www.gsea-msigdb.org/gsea/login.jsp; UCSC Xena: https://xena.ucsc.edu/.

## LIST OF ABBREVIATIONS

LUAD: Lung Adenocarcinoma
Tregs: Regulatory T Cells
TME: Tumor Microenvironment
TRGs: Treg-related Genes
GEO: Gene Expression Omnibus
TCGA: The Cancer Genome Atlas
CNVs: Copy Number Variations
GSVA: Gene Set Variation Analysis
ESTIMATE: Estimation of STromal and Immune cells in MAlignant Tumor tissues using Expression Data
LASSO: Least Absolute Shrinkage and Selection Operator
PCA: Principal Component Analysis
MPR: Major Pathological Response
NMPR: Non-Major Pathological Response
scRNA-seq: Single-cell RNA Sequencing
