# Supplementary material for: Characterization of Tumor Microenvironment and Prognosis of Regulatory T cells-Related Subtypes
Source: Curr Med Chem. 2025 Jul 10;33(4):816–34. doi: 10.2174/0109298673375015250513094405 (PMC13223482; doi:10.2174/0109298673375015250513094405)
Supplement: Supplementary file 1 [file CMC-33-4-816_SD1.pdf]

Supplementary Material

Characterization of Tumor Microenvironment and Prognosis of Regulatory T cells-Related Subtypes

Xinwei Li[1,*], Meiyun Nie[1,*], Keke Yang[1], Xiaodong Qi[1], Xiong Wan[2,3,#] and Ling Yang[1,#]

[1] Department of Geriatrics, Shanghai Fourth People's Hospital affiliated with Tongji University, Shanghai, China; [2]Key Laboratory of Systems Health Science of Zhejiang Province, School of Life Science Hangzhou Institute for Advanced Study, University of Chinese Academy of Sciences, Hangzhou, China; [3]Key Laboratory of Space Active Opto-Electronics Technology of the Chinese Academy of Sciences, Shanghai Institute of Technical Physics, Chinese Academy of Sciences, Shanghai, China

Table S1. Tregs-related gene.

IL12RB2
TMPRSS6
CTSC
LAPTM4B
TFRC
NETO2
CHST2
CTLA4
NFE2L3
LIMA1
IL1R2
ICOS
HSDL2
HTATIP2
CCR8
LTA
SLC35F2
IL21R
AHCYL1
SOCS2
ETV7
BCL2L1
RRAGB
ACSL4
CHRNA6

BATF
LAX1
ADPRH
TNFRSF4
ANKRD10
CASP1
LY75
NPTN
SSTR3
GRSF1
CSF2RB
TMEM184C
ZBTB38
TRAF3
NAB1
HS3ST3B1
JAK1
VDR
LEPROT
GCNT1
PTPRJ
IKZF2
CSF1
ENTPD1
METTL7A
KSR1
SSH1
CADM1
IL1R1
ACP5
CHST7
THADA
CD177
NFAT5
ZNF282
MAGEH1

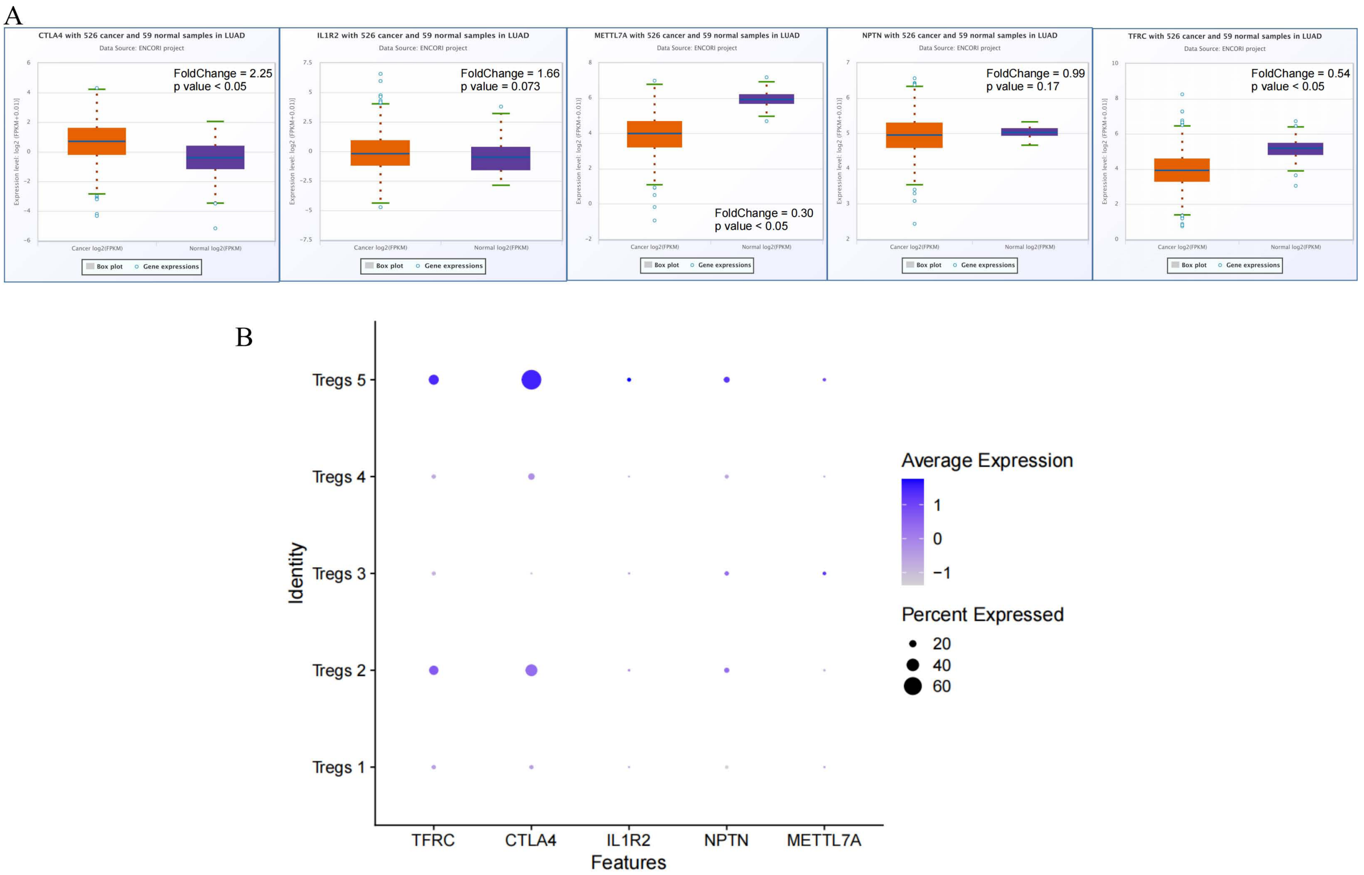

Fig. (S1). (**A**) Expression levels of model genes in the LUAD dataset from the starBase database.(**B**) Differential expression of model genes in Tregs subpopulations from scRNA-seq data.
